# Impact of Uncertainties in Exposure Assessment on Estimates of Thyroid Cancer Risk among Ukrainian Children and Adolescents Exposed from the Chernobyl Accident

**DOI:** 10.1371/journal.pone.0085723

**Published:** 2014-01-29

**Authors:** Mark P. Little, Alexander G. Kukush, Sergii V. Masiuk, Sergiy Shklyar, Raymond J. Carroll, Jay H. Lubin, Deukwoo Kwon, Alina V. Brenner, Mykola D. Tronko, Kiyohiko Mabuchi, Tetiana I. Bogdanova, Maureen Hatch, Lydia B. Zablotska, Valeriy P. Tereshchenko, Evgenia Ostroumova, André C. Bouville, Vladimir Drozdovitch, Mykola I. Chepurny, Lina N. Kovgan, Steven L. Simon, Victor M. Shpak, Ilya A. Likhtarev

**Affiliations:** 1 Radiation Epidemiology Branch, Division of Cancer Epidemiology and Genetics, National Cancer Institute, National Institutes of Health, Department of Health and Human Services, Bethesda, Maryland, United States of America; 2 Ukrainian Radiation Protection Institute, Kyiv, Ukraine; 3 Taras Shevchenko National University of Kyiv, Kyiv, Ukraine; 4 Department of Statistics, Blocker Building, Texas A&M University, College Station, Texas, United States of America; 5 Biostatistics Branch, Division of Cancer Epidemiology and Genetics, National Cancer Institute, National Institutes of Health, Department of Health and Human Services, Bethesda, Maryland, United States of America; 6 Sylvester Comprehensive Cancer Center, University of Miami, Miami, Florida, United States of America; 7 State Institution “Institute of Endocrinology and Metabolism of Academy of Medical Sciences of Ukraine”, Kyiv, Ukraine; 8 Department of Epidemiology and Biostatistics, University of California San Francisco, San Francisco, California, United States of America; Kagoshima University Graduate School of Medical and Dental Sciences, Japan

## Abstract

The 1986 accident at the Chernobyl nuclear power plant remains the most serious nuclear accident in history, and excess thyroid cancers, particularly among those exposed to releases of iodine-131 remain the best-documented sequelae. Failure to take dose-measurement error into account can lead to bias in assessments of dose-response slope. Although risks in the Ukrainian-US thyroid screening study have been previously evaluated, errors in dose assessments have not been addressed hitherto. Dose-response patterns were examined in a thyroid screening prevalence cohort of 13,127 persons aged <18 at the time of the accident who were resident in the most radioactively contaminated regions of Ukraine. We extended earlier analyses in this cohort by adjusting for dose error in the recently developed TD-10 dosimetry. Three methods of statistical correction, via two types of regression calibration, and Monte Carlo maximum-likelihood, were applied to the doses that can be derived from the ratio of thyroid activity to thyroid mass. The two components that make up this ratio have different types of error, Berkson error for thyroid mass and classical error for thyroid activity. The first regression-calibration method yielded estimates of excess odds ratio of 5.78 Gy^−1^ (95% CI 1.92, 27.04), about 7% higher than estimates unadjusted for dose error. The second regression-calibration method gave an excess odds ratio of 4.78 Gy^−1^ (95% CI 1.64, 19.69), about 11% lower than unadjusted analysis. The Monte Carlo maximum-likelihood method produced an excess odds ratio of 4.93 Gy^−1^ (95% CI 1.67, 19.90), about 8% lower than unadjusted analysis. There are borderline-significant (*p = *0.101–0.112) indications of downward curvature in the dose response, allowing for which nearly doubled the low-dose linear coefficient. In conclusion, dose-error adjustment has comparatively modest effects on regression parameters, a consequence of the relatively small errors, of a mixture of Berkson and classical form, associated with thyroid dose assessment.

## Introduction

The accident at the Chernobyl nuclear power plant remains the most serious nuclear accident in history. Thyroid cancer was the first cancer to be elevated among the exposed residents in Ukraine and Belarus, within 5 years of the accident, and the excess is particularly marked among those exposed in childhood [1]–[4]. The thyroid cancer excess is thought to be largely the result of release of radioactive iodine-131 (^131^I) from the Chernobyl reactor.

In collaboration with the Institute of Endocrinology and Metabolism, Kyiv, Ukraine and Columbia University, the U.S. National Cancer Institute initiated a cohort screening study of children and adolescents exposed to Chernobyl fallout in Ukraine to better understand the long-term health effects of exposure to radioactive iodines [5]. Unlike many other studies of thyroid cancer in relation to environmental exposure [6], [7], this cohort incorporates detailed thyroid activity measurements, and mass estimates derived from a similar external Ukrainian sample, crucial to estimates of dose. There have been a number of analyses of this cohort [3], [8], which document the significant increased risk of thyroid cancer in relation to ^131^I thyroid dose. A major source of uncertainty in estimation of low dose risk concerns the extrapolation of risks at high dose and high dose-rates to those at low doses and low dose-rates. Crucial to the resolution of this area of uncertainty is consideration of both systematic and random dosimetric errors in analyses of the Chernobyl-exposed and other exposed groups. The problem of allowing for errors in dose assessments when estimating dose-response relationships has recently been the subject of much research [9]. It is well recognized that measurement error can alter substantially the shape of this relationship and hence the derived study risk estimates [9]. Typically errors are assumed to be of one of two types, classical or Berkson. Classical errors, in which the measured doses are assumed to be distributed with (independent) error around the true dose, generally result in a downward bias of the dose-response parameter [9]. Berkson errors, in which the true dose is randomly distributed around a measured dose estimate, do not result in biased estimates of the dose-response parameter for linear models, although for non-linear models that is not the case [9]. Classical dose errors are generally thought to characterize the errors in dose estimates in the Japanese atomic bomb survivors [10], whereas Berkson errors are thought to dominate the dose uncertainties in certain medical studies [11]. In practice, errors associated with measurement of doses are a mixture of classical and Berkson errors and each type of dose error can include both a shared component, common to all individuals within a group, and an unshared part, unique to an individual within a cohort [12]. Kukush *et al.* [13] developed a novel methodology for assessing dose error in a (simulated) Chernobyl-exposed cohort, incorporating both Berkson errors (relating to thyroid mass estimates), and classical errors (relating to thyroid activity assessments). When dose errors are modest, a commonly used method of dealing with dose error is to replace the dose estimate in any regression with the expected true dose given the measured dose estimate, a process termed regression calibration [9]. When dose uncertainties are more substantial full-likelihood methods may be indicated, in particular Monte Carlo maximum likelihood integration (MCML) [12], [14], and Bayesian Markov Chain Monte Carlo (MCMC) [10].

The dose-response for prevalent thyroid cancers in the Ukrainian-US screening cohort was previously analyzed [3] using the original (TD-02) individual dose estimates, while the dose response for incident thyroid cancer cases was analyzed [8] using a modified version of TD-02, in which adjustments were made to reflect an increased understanding of thyroid mass measurements in the cohort. A further review has resulted in a new set of thyroid dose estimates, referred to as TD-10 [15]. In this paper we assess the impact on thyroid cancer risk of a number of methods of adjustment for the effects of dose uncertainty, in particular regression-calibration and MCML procedures. Most analyses use the TD-10 dosimetry; we also briefly compare our results with those of Tronko *et al.* based on the TD-02 doses [3].

## Data and Methods

### Ethics Statement

The data were hosted at three collaborating institutions: Institute of Endocrinology and Metabolism, Kyiv, Ukraine, Columbia University/University of California San Francisco (UCSF), and the National Cancer Institute (NCI). All subjects signed an informed consent form, and the study was reviewed and approved by the institutional review boards of the participating institutions in both Ukraine and the United States. The data were de-identified before transfer to the United States participating institutions. The key to the data exists in Ukraine, but US researchers did not have access to it at any point. Anonymized data can be provided upon request with conditions agreeable to the three parties (Institute of Endocrinology and Metabolism, Kyiv, Ukraine, Columbia University/UCSF, NCI). At NCI, it has to be formalized through the Technical Transfer Center.

### Study data

The Ukrainian-US prevalence cohort includes 13,127 individuals (44% of the 29,919 potentially available subjects originally selected for the study [3]) who were less than 18 years old on April 26 1986. All cohort members were required to have had at least one direct measurement of thyroid radioactivity between April 30 and June 30, 1986 and to have resided at the time of screening (which was highly correlated with residence at the time of the accident) in the northern areas of Ukraine (Kyiv city and oblast, Zhytomyr, and Chernihiv oblasts), which were the most radioactively contaminated territories in Ukraine as a result of the Chernobyl accident. Thyroid activity measurements were made by means of several types of gamma-counters held against the neck, from which was derived (via subtraction of the background radiation count and other variables) the ^131^I activity in the thyroid gland. For 6 subjects a current (TD-10: see below) thyroid dose could not be estimated; they were excluded from the main analysis cohort for all analyses based on TD-10 doses, but were included for analyses based on TD-02 doses. There were a total of 45 thyroid cancer cases, exactly as in the data of Tronko *et al.* [3].

### Revised dose estimates

The first estimates of individual thyroid doses for all members of the Ukrainian-US cohort were obtained in 2002 (TD-02). Along with a description of the corresponding thyroid dose reconstruction system, the first dose estimates were published by Likhtarev *et al.* [16]. For the second (TD-10) set of thyroid dose estimates [15], [17] the following improvements were carried out:

- A second round of interviews for all cohort members was conducted so that detailed information on personal history (relocation from the contaminated territory and consumption of contaminated foods) could be clarified.
- Parameters of the dosimetry model were substantially improved. They includes estimates of ^131^I ground deposition on the Ukrainian territory using a new mesoscale model of atmospheric transport of the radioactive materials released during the Chernobyl accident; site-specific values of model parameters derived from the available data on radionuclide transport in the environment that were published after the Chernobyl accident; evaluation of the contribution of the incorporated radiocesiums to the signal read by the detectors.
- Oblast-specific thyroid mass estimates were derived using measurements of thyroid volume performed during the 1990s by the Sasakawa Memorial Health Foundation among children and adolescents of Kyiv and Zhytomyr oblasts [18].

The component of the reconstruction model dealing with the input data resulting from direct individual measurement of thyroid activity (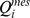) and thyroid mass (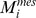) has not been revised; this revision is now underway [19], [20].

### Dose error model

The probabilistic models of thyroid mass and thyroid activity are outlined in Appendix S1. These are applied to the current (TD-10) set of dose estimates. The thyroid mass at the time of the Chernobyl accident was estimated via population-average measurements performed on children aged 5 to 16, taken within a few years of the accident in Kyiv and Zhytomyr oblasts by the Sasakawa Health Memorial foundation [18], supplemented with autopsy measurements conducted on newborns and infants [19]; data for missing ages were obtained via interpolation or extrapolation. The currently available estimates of thyroid mass are those used by Likhtarov *et al.* [15]. The true values of the thyroid mass are determined according to a Berkson measurement error model. For the first regression-calibration method, adapted from Kukush *et al.* [13], Supporting Information expression (S12) is used to determine the likelihood of a given measured dose. For the second regression-calibration method Supporting Information expression (SS12) is used to determine the likelihood of the given activity measurements. The measured activity is associated with a multiplicative classical error model, which is determined by the characteristics of the measuring instrument [55], [56]. The dosimetry estimation system has a stochastic design to model shared errors, and to account for uncertain dose-related parameters. Using that system, certain members of the study team (IAL, VMS) produced 1000 simulations of the posterior distribution of dose to the thyroid for all study subjects. The profile likelihood was then derived by integrating the likelihood over these 1000 dose simulations. The two regression calibration methods are similar, but the second makes slightly stronger assumptions on the independence of certain dosimetric quantities, and *a priori* may be regarded as the less plausible model; however, as noted in Appendix S1, there is little evidence of correlation between thyroid activity and mass of the sort that would invalidate the use of the second model. We judge that it is important to assess the effects of adjusting for dose error using a variety of assumptions and models to determine the sensitivity of results to these assumptions. The geometric standard deviation (GSD) was estimated from individual assessments of measured activity. The models of dose error generate models for the distribution of thyroid dose or activity in these intervals, as detailed in Appendix S1. The results of fitting these models to the dose and activity data for the current (TD-10) dose data via maximum likelihood methods [21] are given in Tables S1 and S2.

### Thyroid cancer risk model

The primary statistical model used was a logistic model of the odds ratio (OR), in which the probability of subject 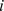 with age at screening 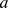, gender 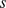, age at exposure 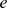 at the time of the accident (1986) and with true thyroid dose:
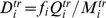
(1)(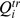 is the true thyroid ^131^I activity in kBq, 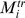 is the true thyroid mass in g, 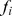 is a scaling constant) being a case of thyroid cancer is given by:



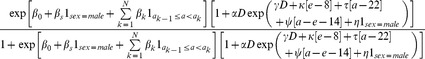
(2)[The age at exposure, 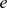, and age at screening, 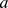, were approximately centered by subtracting off their approximate mean values in the data, namely 8 and 22 years, respectively; this facilitated convergence of the iteratively-reweighted least squares algorithm used to maximize the likelihood [21].] In general only one of the age or temporal adjustment parameters, 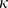, 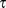 or 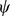 was free to vary. As outlined in Appendix S1, we corrected for the effect of errors in estimates of thyroid activity and mass using two distinct regression calibration approaches and MCML. Using the first regression calibration method, adapted from Kukush *et al.* [13], lead us to substitute 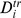 by 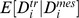 using Supporting Information expression (S16), whereas in the second regression calibration method we substituted 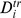 by 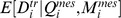 using Supporting Information expression (SS16); these estimates of dose were then substituted in expression (2). All parameters were estimated via maximum likelihood [21]. Appendix S1 also contains further details of the MCML adjustment methods.

## Results

### Comparison of doses

**Figure 1:**
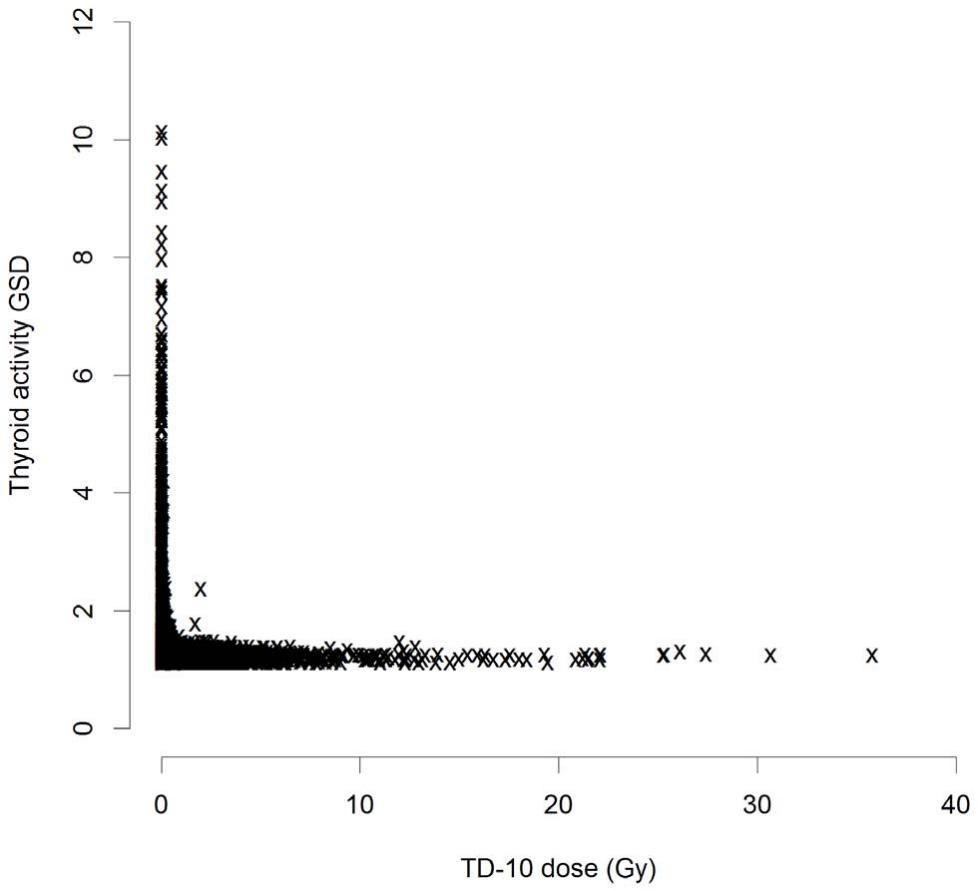
Distribution of the geometric standard deviation (GSD) of errors associated with assessments of thyroid activity GSD as a function of TD-10 thyroid dose. Full dose range.

**Figure 2:**
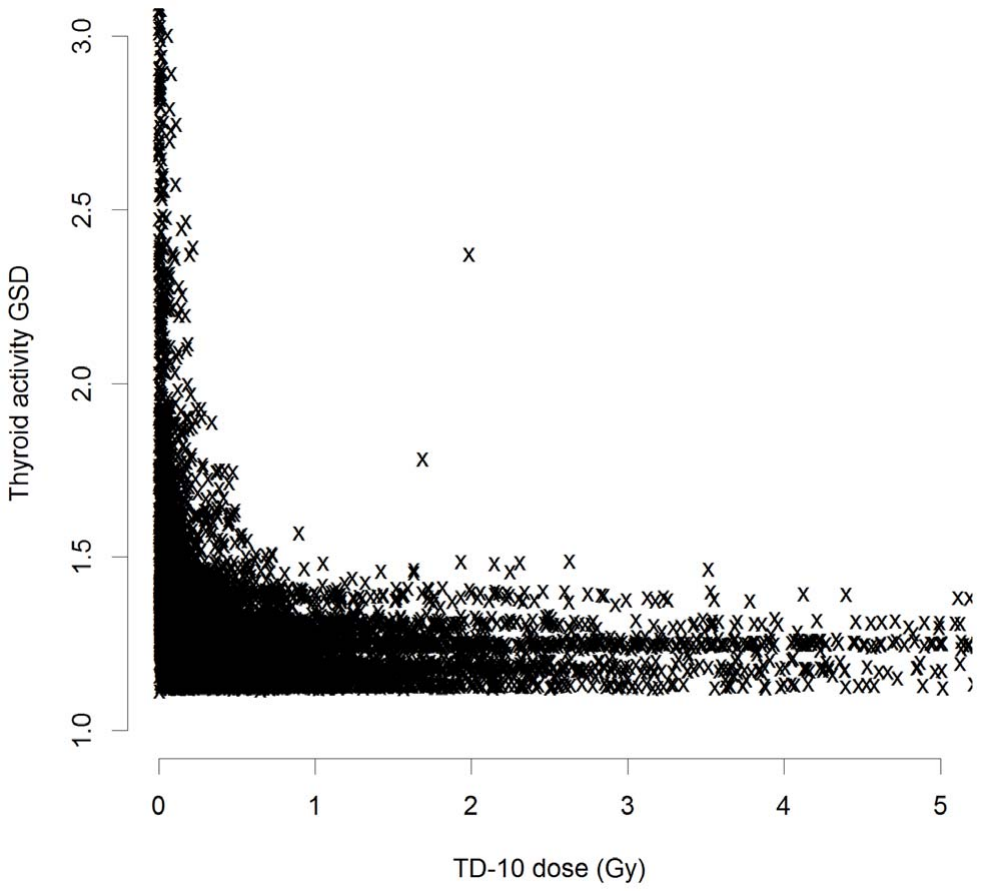
Distribution of the geometric standard deviation (GSD) of errors associated with assessments of thyroid activity GSD as a function of TD-10 thyroid dose. Low dose range.

**Figure 3:**
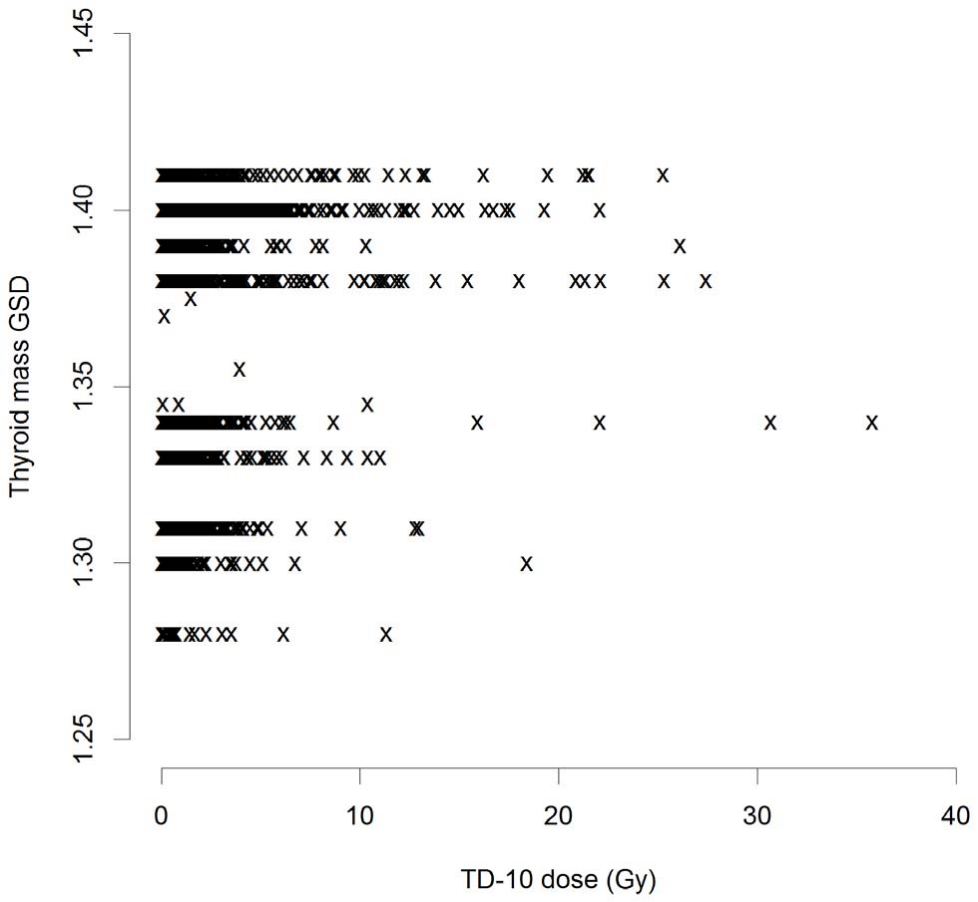
Distribution of the geometric standard deviation (GSD) of errors associated with assessments of thyroid mass GSD as a function of TD-10 thyroid dose.

We found generally good agreement between the TD-02 doses used by Tronko *et al.* [3] and the new (TD-10) dose estimates, although there was considerable scatter (Figures S1, S2). Figure S3 demonstrates that the dose is distributed very-nearly log-normally. The details of the distribution of the GSD associated with errors in the assessments of thyroid activity and mass are given in Table 1; they are shown as a function of TD-10 dose in Figures 1–3. The thyroid activity GSD cover a wide range, 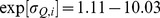, although apart from a wide scatter at lower dose (<0.5 Gy), they are mostly under 1.5, with a mean 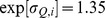. The variation in thyroid mass GSD is generally even less than this (Figure 3), with a range of 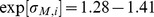 and a mean of 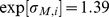 (Table 1).

**Table 1:** Distribution of the geometric standard deviation (GSD) of errors associated with measurements of thyroid activity and of thyroid mass across individuals within the cohort.

TD-10 dose range (Gy)	Range	Mean	Median	10%, 90%
Thyroid activity GSD ( 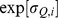 )				
0–0.5	1.11–10.13	1.39	1.29	1.16, 1.56
>0.5–1.0	1.12–1.57	1.23	1.22	1.13, 1.33
>1.0–5.0	1.12–2.37	1.23	1.22	1.13, 1.31
>5.0–10.0	1.12–1.40	1.23	1.25	1.13, 1.29
>10.0	1.13–1.47	1.21	1.19	1.17, 1.26
Total	1.11–10.13	1.35	1.26	1.16, 1.49
Thyroid mass GSD ( 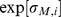 )				
0–0.5	1.28–1.41	1.39	1.40	1.34, 1.40
>0.5–1.0	1.28–1.41	1.39	1.40	1.33, 1.40
>1.0–5.0	1.28–1.41	1.38	1.40	1.33, 1.40
>5.0–10.0	1.28–1.41	1.39	1.40	1.33, 1.41
>10.0	1.28–1.41	1.38	1.39	1.34, 1.41
Total	1.28–1.41	1.39	1.40	1.34, 1.40

### Model fitting

#### Comparison of effects of various adjustments for dose error in logistic model

**Figure 4:**
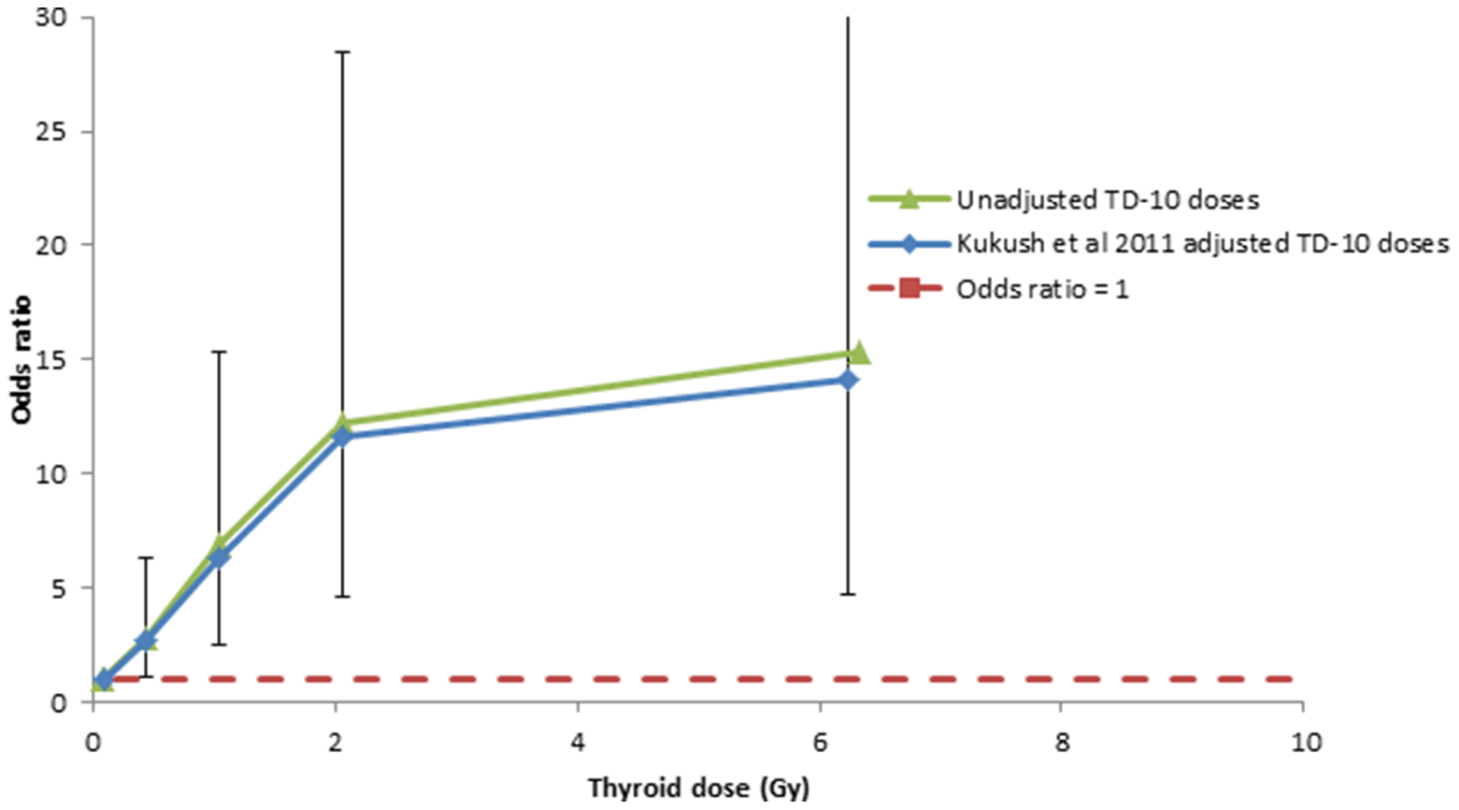
Dose response (+95 CI) for thyroid cancer in relation to TD-10 unadjusted dose, and regression-calibration-adjusted dose (using 1^st^ method, adapted from Kukush *et al.* [13]). The models are adjusted for age (treated categorically) and gender in the baseline. Dashed red line shows odds ratio  = 1.

Table 2 demonstrates that using the logistic model (2), there is a highly statistically significant increasing dose response (*p*<0.001) for all four sets of dose estimates and models (TD02, unadjusted current (TD-10), current (TD-10) + first/second type of regression-calibration adjustments and MCML). The dose response using the first regression calibration method, adapted from Kukush *et al.* [13], is shown in Figure 4, as also the unadjusted dose response for comparison. Table 2 demonstrates that without adjustment for dose errors the EOR was about 2% higher with the TD-10 doses, 5.38 Gy^−1^ (95% CI 1.86, 21.01), than with the TD-02 doses, 5.25 Gy^−1^ (95% CI 1.70, 27.45). The first regression-calibration method, adapted from Kukush *et al.* [13], yielded estimates of the EOR of 5.78 Gy^−1^ (95% CI 1.92, 27.04), about 7% higher than estimates unadjusted for dose error. The second regression-calibration method yielded an EOR of 4.78 Gy^−1^ (95% CI 1.64, 19.69), about 11% lower than TD10 estimates unadjusted for dose error. The MCML method yielded an EOR of 4.93 Gy^−1^ (95% CI 1.67, 19.90), about 8% lower than the unadjusted TD10 dose estimates.

**Table 2:** Analysis of curvature in fits of EOR model (2) with or without adjustment for dose errors using regression calibration, for TD-10 doses.

Dose	Dose-response model	*p*-value^a^	Linear ERR (*α*) (Gy^−1^) (+95% CI)	Exponential ERR (γ) (Gy^−1^) (+95% CI)
Tronko *et al.* (TD-02) dose	*αD*	<0.001^b^	5.25 (1.70, 27.45)	
Tronko *et al.* (TD-02) dose	*αD *exp[γ*D*]	0.084	9.13 (2.46, 111.1)	−0.09 (−0.23, 0.01)
TD-10 unadjusted dose	*αD*	<0.001^b^	5.38 (1.86, 21.01)	
TD-10 unadjusted dose	*αD *exp[γ*D*]	0.104	8.85 (2.60, 54.58)	−0.11 (−0.29, 0.02)
1^st^ regression calibration method (Kukush *et al.*) adjusted dose	*αD*	<0.001^b^	5.78 (1.92, 27.04)	
1^st^ regression calibration method (Kukush *et al.*) adjusted dose	*αD *exp[γ*D*]	0.112	9.72 (2.67, 94.31)	−0.10 (−0.28, 0.02)
2^nd^ regression-calibration method adjusted dose	*αD*	<0.001^b^	4.78 (1.64, 19.69)	
2^nd^ regression-calibration method adjusted dose	*αD *exp[γ*D*]	0.101	8.19 (2.33, 60.87)	−0.09 (−0.25, 0.02)
Monte Carlo maximum likelihood	*αD*	<0.001^b^	4.93 (1.67, 19.90)	
	*αD *exp[γ*D*]	0.102	7.97 (2.32, 49.81)	−0.09 (−0.26, 0.01)

All models have underlying rates adjusted for age (treated categorically) and gender. Unless otherwise stated all CI are profile-likelihood based.

a unless otherwise stated all *p*-values refer to the improvement in fit of the current row in the Table with that of the model fitted in the row immediately above.

b *p*-value of improvement in fit compared with a model with no dose terms.

Table 2 demonstrates that there were borderline significant indications of downward curvature in the dose response (e.g., *p* = 0.112 for curvature assessed using the first set of regression-calibration-adjusted doses). The effect of allowing for this was to nearly double the linear coefficient, from 5.78 Gy^−1^ (95% CI 1.92, 27.04), to 9.72 Gy^−1^ (95% CI 2.67, 94.31). However, the effect of adjustment for dose error on the coefficients of the indicated linear-exponential model were not much more substantial than for the linear model. For example the linear coefficient of a linear-exponential model without dose-error adjustment was 8.85 Gy^−1^ (95% CI 2.60, 54.58), and after adjustment using the first regression calibration method, adapted from Kukush *et al.* [13], this became 9.72 Gy^−1^ (95% CI 2.67, 94.31), an increase of 10%; after adjustment using the second regression calibration method this became 8.19 Gy^−1^ (95% CI 2.33, 60.87), a decrease of 7%.

**Figure 5:**
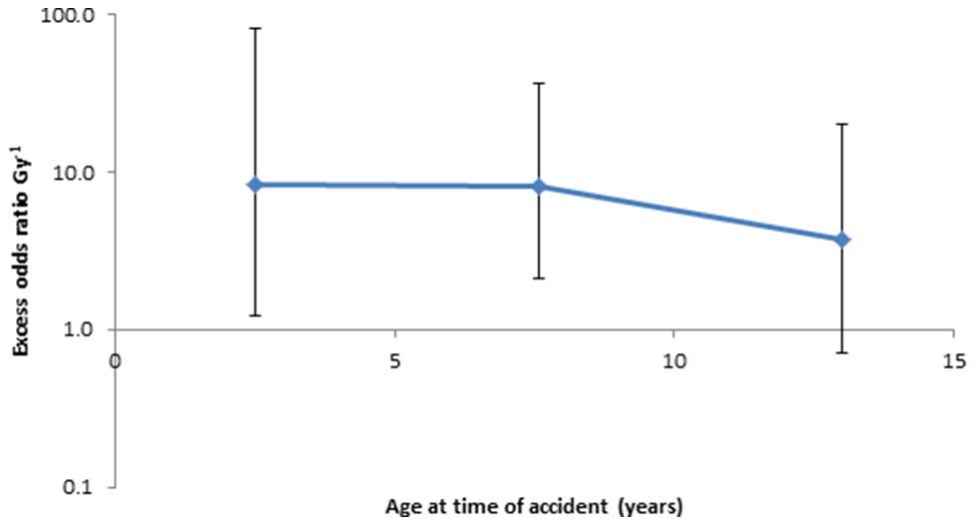
Variation of excess relative risk with age at the time of the accident (using 1^st^ regression calibration method, adapted from Kukush *et al.* [13]). Other details as for Figure 4.

Table 3 demonstrates that the modifying effects of gender, age at the time of the accident, age at screening and time since the accident as modifiers of the radiation dose response were generally not statistically significant (*p*>0.1) (see also Figure 5); this is the case whichever set of dose estimates are employed (results not shown). Table S3 reports the results of sensitivity analyses, in which certain variables were added to the background model, and does not suggest that any improved the fit over age and sex (*p*≥0.1), nor was there generally any effect on EOR.

**Table 3:** Results of fits of optimal excess relative risk model (2) (maximum likelihood fits and 95% profile CI), all based on TD-10 dose estimates adjusted using 1^st^ regression calibration method (of Kukush *et al.*). All models have underlying rates adjusted for age (treated categorically) and gender. Parameters are given (with 95% CI), with associated *p*-values.^a^ Unless otherwise stated all CI are profile-likelihood based.

Modelnumber	Form ofexcess oddsratio model	Parameters	Estimates(+95% CI)and *p*-values	*p*-value
1	*αD*	*α* (Gy^−1^)	5.78(1.92,27.04)	<0.001^b^
2	*αD*exp[*γD*]	*α* (Gy^−1^)	9.72(2.67,94.31)	0.112
		*γ* (Gy^−1^)	−0.10(−0.28,0.02)	
3	*αD*exp[*γD* +*κ*(*e* –8)]	*α* (Gy^−1^)	12.54(3.33,73.93)	0.161
		*γ* (Gy^−1^)	−0.11(−0.28,0.01)	
		*κ* (years^−1^)	−0.14(−0.37,0.06)	
4	*αD*exp[*γD* +*τ*(*a* –22)]	*α* (Gy^−1^)	11.46(3.17,62.58)	0.172^c^
		*γ* (Gy^−1^)	−0.11(−0.28,0.01)	
		*τ* (years^−1^)	−0.14(−0.37,0.06)	
5	*αD*exp[*γD* +*ψ*(*a* – *e* –14)]	*α* (Gy^−1^)	10.09(2.58,134.60)	0.874^c^
		*γ* (Gy^−1^)	−0.10(−0.28,0.02)	
		*ψ* (years^−1^)	0.04(−0.51,0.59)	
6	*αD*exp[*γD* +*η*1_sex = male_]^d^	*α* (Gy^−1^)	40.44(−119.7^e^,200.6^e^)	0.171c d
		*γ* (Gy^−1^)	−0.12(−0.30,0.01)	
		*η* (years^−1^)	−2.21(−6.47^e^,2.05^e^)	

a Unless otherwise stated all *p*-values refer to improvement in fit of model immediately above indicated one in the Table.

b *p*-value for improvement in fit over null model (without linear dose term).

c *p*-value for improvement in fit over model 2, linear-exponential in dose.

d indications of lack of convergence.

e Wald-based CI.

## Discussion

Re-analysis of the latest follow-up of the Ukrainian-US thyroid prevalence screening study, and using the most current (TD-10) set of dose estimates, demonstrates that there is a highly statistically significant increasing dose response (*p*<0.001), confirming the results of an earlier analysis of this dataset [3]. Adjustment of the regression for dose errors yielded little change in radiation risk estimates, as also did the change from the older (TD-02) to the newer (TD-10) dosimetry.

A major source of uncertainty in estimates of low dose cancer risk concerns the extrapolation of risks at high dose and high dose-rates to those at low doses and low dose-rates. Crucial to the resolution of this area of uncertainty is the flexible modeling of the dose-response relationship and the importance of both systematic and random dosimetric errors. The problem of allowing for errors in dose assessments when estimating dose-response relationships has recently been the subject of much interest in epidemiology [9]. It is well recognized that measurement error can alter substantially the shape of this relationship [22]. Much work has been carried out on assessing the impact of dosimetric error for Japanese atomic bomb survivor data. In particular, Pierce *et al.* [23], [24] carried out a dose adjustment prior to the model fitting, allowing for random dosimetric errors. A similar procedure was followed by Little *et al.* [25]–[28]. This dose adjustment entails the substitution of the “estimated dose” by the expectation of the “true dose” given the estimated one. This approach to measurement error correction is an example of regression calibration, which as emphasized by Carroll *et al.* [9], is an approximate method in non-linear dose-effect relationships. It leads to reasonable adjusted point estimates of the model parameters but does not fully take account of all the variability induced by the measurement errors.

A Bayesian approach to the measurement-error problem has been developed [29]–[31] which rests on the formulation of conditional independence relationships between different model components, following the general structure outlined by Clayton [32]. In this approach three basic sub-models are distinguished and linked: the disease model, the measurement model and the exposure model. The general advantage of Bayesian methods, and other techniques based on use of the full likelihood such as Monte-Carlo Maximum Likelihood (MCML) [14] is that they take full account of the impact of dose errors on regression estimates. An adapted Bayesian method of correction for measurement error – the two-stage Bayesian method – has been applied to the fitting of generalized relative risk models to the Japanese atomic bomb survivor cancer mortality data [10], [33]–[35].

Bayesian methods offer ways of taking account both of dosimetric uncertainties and modeling ones, for example in the form and shape of the dose response and in temporal and age trends. Bayesian Markov Chain Monte Carlo (MCMC) techniques have previously been much used to assess uncertainties in radiation risk [10], [33]–[35]. Bayesian MCMC approaches have the particular advantage that one has an arbitrarily large collection of realizations of model parameters sampled from the posterior distribution, so that uncertainty in any function of these parameters, for example various measures of lifetime population risk, can be directly evaluated by applying the function to the posterior chain sample [10], [33]–[35]. More limited assessment of modeling uncertainties can also be dealt with using multi-model inference (MMI) [36], [37]. MMI methods have also been used in radiation epidemiology [38]–[40]. Although not explicitly Bayesian, MMI is somewhat related to Bayesian model-averaging and related Bayesian techniques [41]; these Bayesian methods have the advantage of assessing the parameter uncertainty distribution more thoroughly than MMI, albeit at somewhat greater computational cost. However, in general Bayesian MCMC and other full-likelihood methods such as MCML, employed here, offer a more flexible and powerful framework for assessing dosimetric and modeling uncertainty than MMI.

In the present case dose errors were modest, particularly at the higher doses that will largely drive the trends with dose (Table 1, Figures 1–3), so that regression calibration methods are likely to be adequate [9], as confirmed by the results obtained using MCML – the results of this latter method is close to those obtained using either of the regression calibration methods, particularly the second. The two regression-calibration methods we used for adjusting for dose errors are similar, but the second makes slightly stronger assumptions on the independence of certain dosimetric quantities, and *a priori* may be regarded as the less plausible model; however, as noted in Appendix S1, there is little evidence of correlation between thyroid activity and mass of the sort that would invalidate the use of the second model. Unusually, both methods take account of mixed Berkson and classical errors in dose, arising from the distinct measurement and estimation associated with thyroid mass and ^131^I thyroid activity measurements. However, neither method makes appreciable difference to regression risk estimates, the first method leading to a 8% increase in EOR, the second an 11% decrease, while the MCML method results in a 8% decrease in EOR, changes which are clearly minimal in relation to the substantial uncertainties (Table 2). The reasons for the relatively modest impact of adjusting for dose error are very largely a consequence of the fact that the errors relating to the thyroid mass, are Berksonian, and as such would not be expected to modify risk estimates [9], [42], but that in any case both these and the classical errors associated with measurements of thyroid activity are relatively small (Table 1, Figures 1–3). Besides the presence of Berkson measurement error, another possible reason for the slightly different adjustments to the unadjusted risks between the two regression-calibration methods is that within the first such method, there is no assumption about independency of true activity, 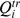, and measured thyroid gland mass, 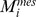, whereas the other method relies on this assumption.

While it is generally to be expected that correction for the effects of measurement error, particularly classical error, will be to increase risks, this is not necessarily the case when, as here, errors are modest (Table 1, Figures 1–3) and part of the error is of Berkson type. In particular Schafer *et al.* [11] document a 8–13% reduction in risks after adjustment for dose measurement errors in a study of thyroid cancer in a group of Israeli children treated for tinea capitis; the errors in this study were largely Berkson. In a study of effects of air-pollution on lung function in a group of Southern California children, adjusting for errors in position (which were largely classical) led to a reduction of effect [43]. More generally, it is known that non-differential misclassification of exposure can bias risks away from the null, or induce a change in sign of a regression trend [44].

The prevalence excess odds ratio that we derived of 5.78 Gy^−1^ (95% CI 1.92, 27.04) using the first regression-calibration method (Table 2) is somewhat higher than, but statistically consistent with that which can be derived from the Japanese atomic bomb survivors exposed to external radiation under the age of 20, 3.07 Gy^−1^ (90% CI 2.14, 4.14) [35]. It is lower than (and again statistically compatible with) the estimate of 7.7 Gy^−1^ (95% CI 2.1, 28.7) derived from a pooled analysis of five childhood-exposed groups [45]. However, the analyses of UNSCEAR [35] and Ron *et al.* [45] are based on incidence data, and the interpretation is therefore somewhat different from the prevalence risk that we estimate. Ron *et al.* [45] also computed a pooled ERR/Gy allowing for a non-zero ERR at zero dose (essentially allowing for an additional offset in risk independent of radiation dose), which was 3.8 Gy^−1^ (95% CI 1.4, 10.7) [45].

An additional consideration in comparing risks derived here with low-dose risk coefficients assessed elsewhere is the substantial uncertainty in the shape of the dose response (in this cohort and others), and the implied uncertainties this introduces into the extrapolated low-dose risk. As was previously found using the older (TD-02) dosimetry [3], we observed borderline significant downward curvature (in other words, a progressive reduction with increasing dose in the upward slope of ERR, rather than negative slope) in the dose response (*p* = 0.101–0.112, Table 2), as shown in Figure 4. The effect of allowing for this was to nearly double the low-dose linear coefficient, from 5.78 Gy^−1^ (95% CI 1.92, 27.04), to 9.72 Gy^−1^ (95% CI 2.67, 94.31) (Table 2). The thyroid is known to be one of the most radiosensitive organs [35], in particular there is abundant literature documenting excess thyroid cancer after exposure to external radiation in childhood [45]. The pooled analysis of Ron *et al.* [45] indicated that in general thyroid cancer exhibited a linear dose response, with indications of a reduction of risk at high doses (>20 Gy). However, Zablotska *et al.* observed a similar reduction to ours in risk above 5 Gy in a cohort of Chernobyl-exposed children and adolescents in Belarus [4]. Cardis *et al.* also observed a turnover in dose response above about 5 Gy in a case-control study of Chernobyl-exposed children in Belarus and the Russian Federation [7]. Sigurdson *et al.* [46] observed a reduction in the thyroid cancer dose response, although at a much higher dose, of about 20 Gy, in a group followed after treatment with radiotherapy for cancer in childhood. As such, the turnover that we, Zablotska *et al.* [4] and Cardis *et al.* [7] observe, is reasonably quantitatively consistent. It is possible that this downturn reflects the effect of cell sterilization, a well-known phenomenon in radiobiology and radio-epidemiology [47], and which has been modelled in various other endpoints [48]-[50]. The magnitude of the exponential coefficient, 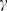, that we obtain is between −0.11 Gy^−1^ and −0.09 Gy^−1^ (Table 2). Deschavanne and Fertil [51] surveyed 42 *in vitro* studies that assessed 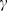 for a variety of fibroblastic and other human cell lines, with values ranging from −1.72 Gy^−1^ to −0.30 Gy^−1^, and a median value of −0.65 Gy^−1^. As such, our value looks a little too small (too near 0). However, there may be compensating tissue repopulation during the course of exposure to ^131^I from Chernobyl, which would be expected to substantially reduce the observed value of 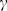 [52].

There were no strong indications (*p*>0.1) of variation of relative risk with age at exposure, age at screening, or time since exposure (Table 3). There is considerable evidence that thyroid cancer relative risk decreases with increasing age at exposure [35], [45]; it is not altogether obvious why this was observed only relatively weakly here (Figure 5). There are weaker indications of eventual reductions of thyroid cancer relative risk with increasing time after exposure among those exposed in childhood [45], [53], [54]. It is likely that our cohort, with follow-up confined to a relatively narrow time interval, 1998–2000, about 12–14 years after the Chernobyl accident, lacks the power to detect such downturns in risk, which in any case would not be expected until 15–19 years after the accident [45].

## Conclusions

The results of the paper are based on a screening study of the most heavily exposed populations in Ukraine who were aged under 18 years old at the time of the Chernobyl accident. The paper extends previous analyses by using revised thyroid cancer dose estimates. This paper addresses for the first time the errors that are present in absorbed thyroid doses, and their effect on thyroid cancer risk estimates; however, the effects of adjusting for dose error are minimal, resulting in changes to cancer risk estimates by between −11% and +7%. In relation to the other uncertainties in the data, these relatively modest changes in risk resulting from taking dose errors into account are largely a consequence of the modest size of the errors and the fact that a component (associated with measurement of thyroid mass) is of Berkson type. There is borderline statistically significant reduction in the upward slope of thyroid cancer risk at high doses.

## Supporting Information

Figure S1 **Comparison of TD-02** [**3**] **and current (TD-10) dose estimates.** Current (TD-10) dose *vs* TD-02 dose.
(TIFF)

Figure S2 **Comparison of TD-02** [**3**] **and current (TD-10) dose estimates.** Current (TD-10) dose *vs* TD-02 dose. Ratio current (TD-10) dose:TD-02 dose *vs* TD-02 dose.
(TIFF)

Figure S3 **Quantile-quantile plot for ln[current (TD-10) dose] data.**
(TIFF)

Appendix S1 **Supporting Information.** Dosimetric error model and other statistical details.
(DOCX)

Table S1 **Fits of multiple log-normal models as given by expression (S12) to measured dose data (1^st^ regression calibration model).**
(DOCX)

Table S2 **Fits of multiple log-normal models to measured activity data (2^nd^ regression calibration model).**
(DOCX)

Table S3 **Effect of additional background variables on thyroid cancer prevalence risk (EOR/Gy).** All use a linear EOR model, with regression calibration dose adjustments adapted from Kukush *et al* [13]. All CI are profile-likelihood based.
(DOCX)
