# Supplementary figures and images for: Impact of Uncertainties in Exposure Assessment on Estimates of Thyroid Cancer Risk among Ukrainian Children and Adolescents Exposed from the Chernobyl Accident
Source: PLoS One. 2014 Jan 29;9(1):e85723. doi: 10.1371/journal.pone.0085723 (PMC3906013; doi:10.1371/journal.pone.0085723)

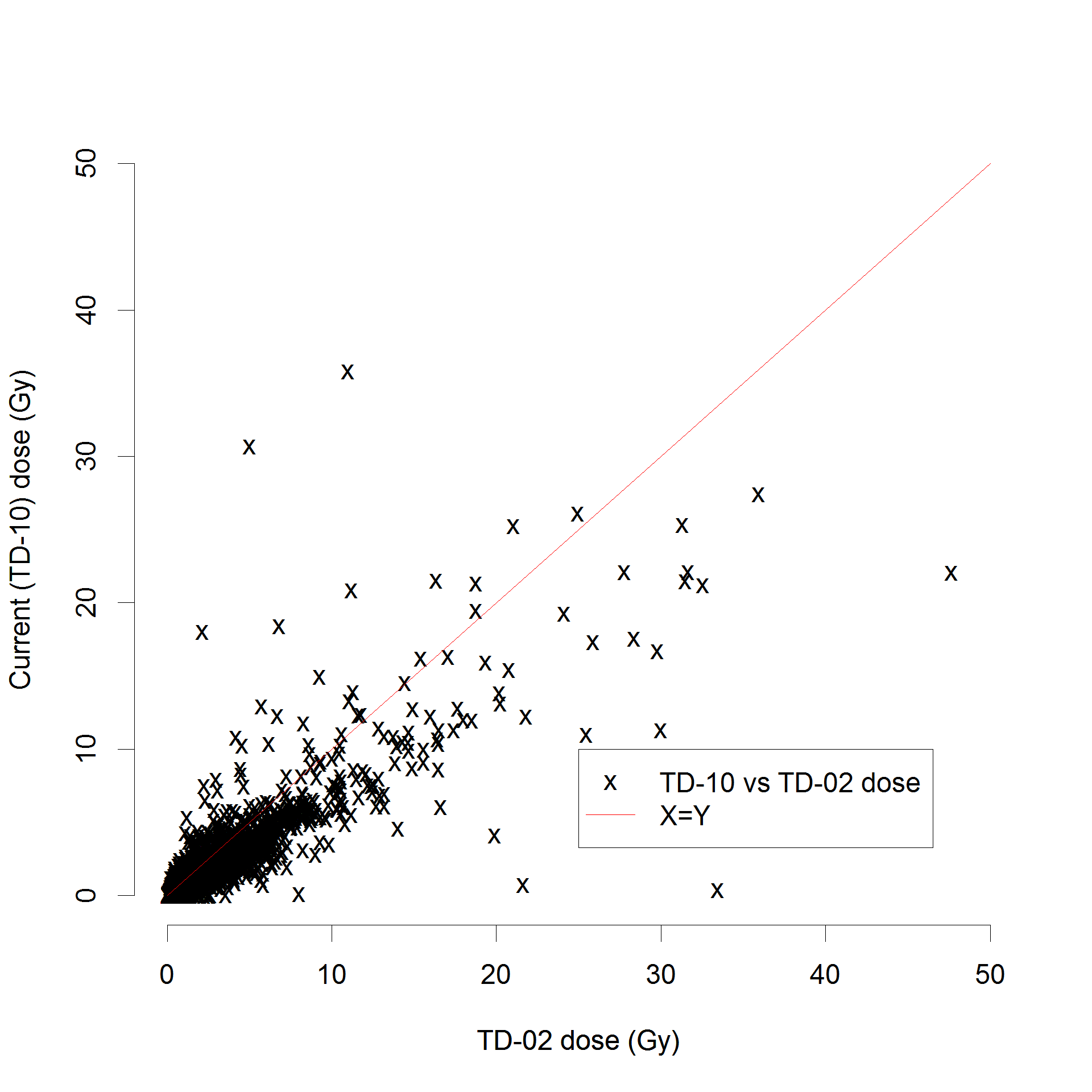

Supplement: Figure S1 — Comparison of TD-02 [3] and current (TD-10) dose estimates. Current (TD-10) dose vs TD-02 dose. (TIFF) [file pone.0085723.s001.tiff]

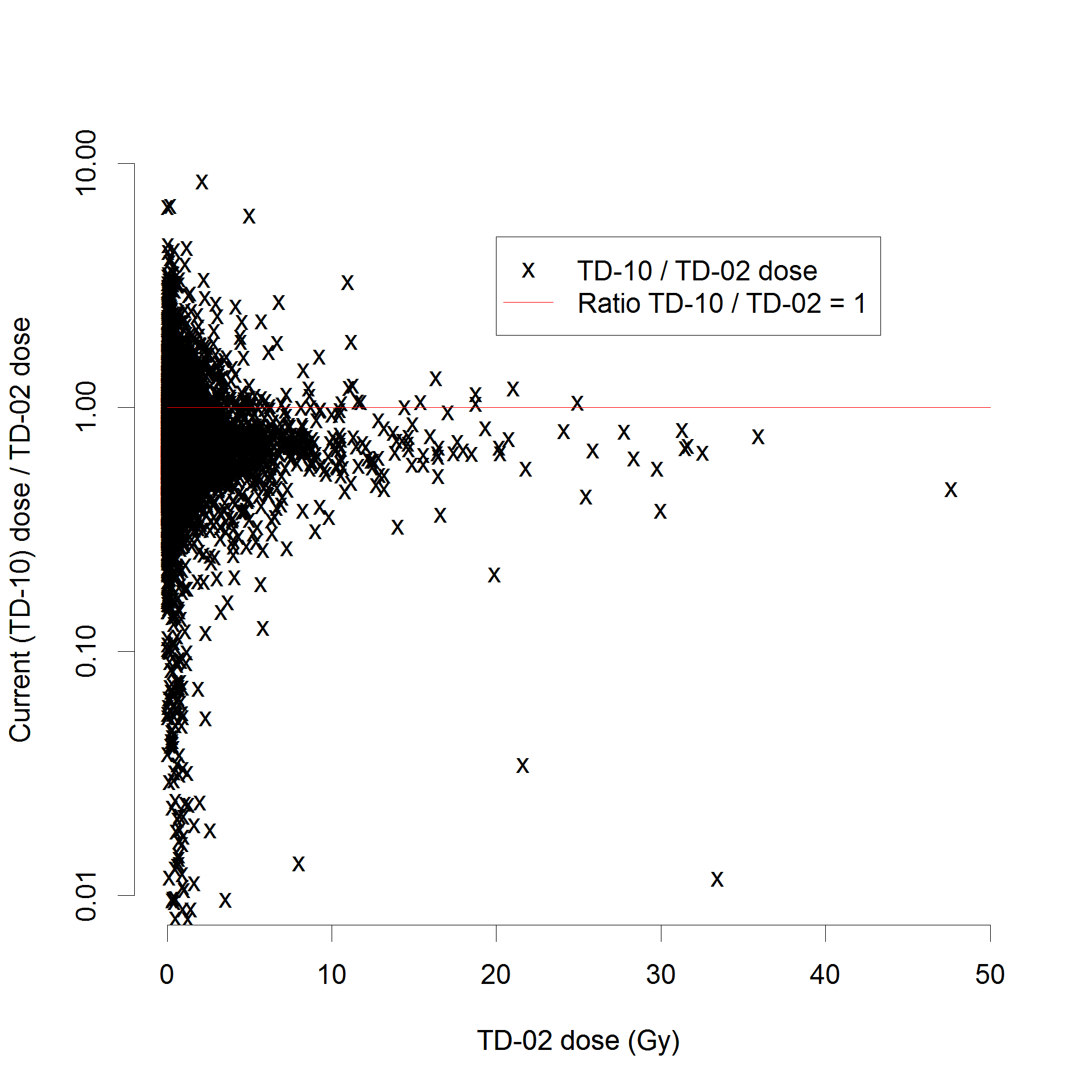

Supplement: Figure S2 — Comparison of TD-02 [3] and current (TD-10) dose estimates. Current (TD-10) dose vs TD-02 dose. Ratio current (TD-10) dose:TD-02 dose vs TD-02 dose. (TIFF) [file pone.0085723.s002.tiff]

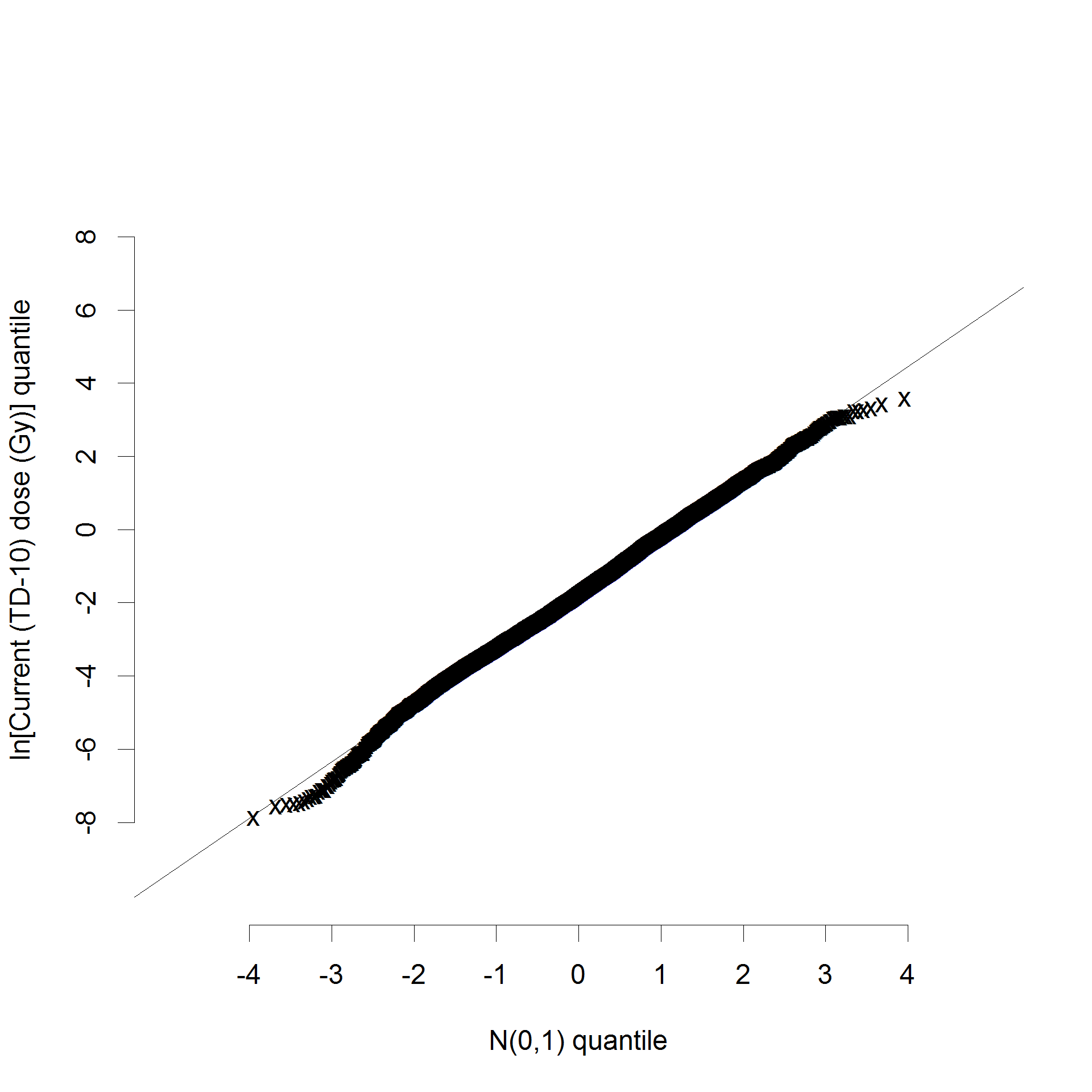

Supplement: Figure S3 — Quantile-quantile plot for ln[current (TD-10) dose] data. (TIFF) [file pone.0085723.s003.tiff]
