# Supplementary material for: Impact of Uncertainties in Exposure Assessment on Estimates of Thyroid Cancer Risk among Ukrainian Children and Adolescents Exposed from the Chernobyl Accident
Source: PLoS One. 2014 Jan 29;9(1):e85723. doi: 10.1371/journal.pone.0085723 (PMC3906013; doi:10.1371/journal.pone.0085723)
Supplement: Appendix S1 — Supporting Information. Dosimetric error model and other statistical details. (DOCX) [file pone.0085723.s004.docx]

**Appendix S1. Supporting Information. Dosimetric error model and other statistical details**

In this Appendix we outline the two regression calibration methods for dose error correction used in the paper. The first is an adaptation of the method presented in the paper of Kukush *et al.* [13] for dealing with dose measurement error in the Ukrainian-US Thyroid Screening Study. The method outlined differs from that of Kukush *et al.* [13] in using a sum of log-normal distributions to model the underlying quasi-true dose distribution rather than the single log-normal (or piecewise-constant) distribution assumed by Kukush *et al.* [13]. We contrast this with a very similar method that makes slightly more stringent distributional assumptions. In the main paper we present results of fitting the latest (2010) Ukrainian-US data using these two types of regression-calibration adjustment. In the equations that follow we distinguish those equations corresponding exclusively to the second method (SS1), (SS2), ..., to distinguish them from (S1), (S2), ..., corresponding to the adapted method of Kukush *et al.* [13] or the second method where this does not differ.

According to the dosimetric model described in Likhtarev *et al.* [16], the calculated thyroid dose of person $i$ is expressed as:

$D_{i}^{ins}=f_{i}\frac{Q_{i}^{mes}}{M_{i}^{est}}$ (S1)

where $Q_{i}^{mes}$ is the measured content of $^{131}\text{I}$ in the thyroid gland of person $i$ at time $t_{mes}$, $M_{i}^{est}$ is the estimate of the thyroid mass, and $f_{i}$ is a multiplier which takes into account the parameter values of an ecology-metabolism model for person $i$. While $f_{i}$ is also measured with error, empirical evaluations of its variation suggest that its error is for most individuals much smaller than the errors in $Q_{i}^{mes}$ and in $M_{i}^{est}$ (see Likhtarev *et al.* [16]) and we shall ignore this component in the analysis. In principle, the uncertainty in $f_{i}$ can be included in the evaluation in many ways, using, for example, the methods of Stram and Kopecky [12]. It is known that most of the uncertainty in $f_{i}$ is associated with unshared error, the result of individual behavior of the cohort members. We outline a method similar in principle to the likelihood integration method of Fearn *et al.* [14], which was developed with application to the residential radon case-control studies. It has elements in common with the method of Stram and Kopecky [12].

The uncertainties in $Q_{i}^{mes}$ and $M_{i}^{est}$are as follows:

- The measured activity is associated with a multiplicative error, which is determined by the characteristics of the measuring instrument [55,56], so that:

$Q_{i}^{mes}=Q_{i}^{tr}V_{i}^{Q}$ (S2)

where $Q_{i}^{tr}$ is the true $^{131}\text{I}$ content in the thyroid gland, and $V_{i}^{Q}$ is the independent multiplicative measurement error. If we assume that $V_{i}^{Q}$ is log-normally distributed, so that $\ln(V_{i}^{Q})\sim N(0,\sigma_{Q,i}^{2})$, then the conditional pdf $Q_{i}^{mes}|Q_{i}^{tr}$ is:

$P[Q_{i}^{mes}|Q_{i}^{tr}]=\frac{1}{\sigma_{Q,i} \sqrt{2\pi}Q_{i}^{mes}}\text{exp}\left[-\frac{[\ln(Q_{i}^{mes})-\ln(Q_{i}^{tr})]^{2}}{2\sigma_{Q,i}^{2}}\right]$ (S3)

Expressions (S2)-(S3) of course define a classical multiplicative error model.

- The true values of the thyroid mass $M_{i}^{tr}$ are determined according to a Berkson measurement error model as:

$M_{i}^{tr}=M_{i}^{mes}V_{i}^{M}$ (S4)

where $M_{i}^{mes}$ is the median of the thyroid mass for a given gender-age group, and $V_{i}^{M}$ an independent multiplicative error. The measured median thyroid mass values, $M_{i}^{mes}$, by age and oblast, are those used by Likhtarev *et al.* [15]. If we assume that $V_{i}^{M}$ is log-normally distributed, so that $\ln(V_{i}^{M})\sim N(0,\sigma_{M,i}^{2})$, then the conditional pdf $M_{i}^{tr}|M_{i}^{mes}$is:

$P[M_{i}^{tr}|M_{i}^{mes}]=\frac{1}{ \sigma_{M,i}\sqrt{2\pi}M_{i}^{tr}}\text{exp}\left[-\frac{[\ln(M_{i}^{tr})-\ln(M_{i}^{mes})]^{2}}{2\sigma_{M,i}^{2}}\right]$ (S5)

From this it follows that:

$E\left[\left.\frac{1}{M_{i}^{tr}}\right|M_{i}^{mes}\right]=E\left[\left.\frac{1}{M_{i}^{mes}V_{i}^{M}}\right|M_{i}^{mes}\right]=\frac{1}{M_{i}^{mes}}E\left[\left.\frac{1}{V_{i}^{M}}\right|M_{i}^{mes}\right]=\frac{1}{M_{i}^{mes}}\exp\left[\frac{\sigma_{M,i}^{2}}{2}\right]$ (S6)

As in Kukush *et al.* [13] we assume that the random variables $Q_{i}^{tr}/M_{i}^{mes}$ and $V_{i}^{Q}$ and $V_{i}^{M}$ are jointly independent, and we define $M_{i}^{est}=M_{i}^{mes}$. This is strictly weaker than the assumption made by the US Working Group (USWG), that the random variables $(Q_{i}^{tr},V_{i}^{Q},M_{i}^{mes},V_{i}^{M})$ are jointly independent. In the Ukrainian-US study, the parameters $\sigma_{Q, i}^{2}$ and $\sigma_{M, i }^{2}$ have been reasonably reliably estimated from various sources [16] (although this is not the case for thyroid mass measurements outside the age range 5-15, for which more or less subjective assignments were made), and therefore we assume that these variables are known.

The (unobservable) error-free dose is denoted by $D_{i}^{tr}$ and is given by:

$D_{i}^{tr}=f_{i}Q_{i}^{tr}/M_{i}^{tr}$ (S7)

Based on the analysis of real samples taken from an epidemiological study [16], we provisionally assume that the logarithm of the quasi-true dose $Y_{i}=f_{i}Q_{i}^{tr}/M_{i}^{mes}$ (it is not the true dose because of the presence of $M_{i}^{mes}$ rather than $M_{i}^{tr}$) as a distribution which can be represented by a mixture of $K$ normal distributions, and hence can be written as:

$\text{ln}(f_{i}Q_{i}^{tr}/M_{i}^{mes})\sim\sum_{k=1}^{K} p_{k}N(\ln(\mu_{k}), \sigma_{k}^{2})$ (S8)

where $\sum_{k=1}^{K} p_{k}=1$. It should be noted that in general all the parameters, in particular $K$, $(p_{k})_{k=1}^{K}$, $(\mu_{k})_{k=1}^{K}$. $(\sigma_{k}^{2})_{k=1}^{K}$ are unknown, and must be estimated. We outline a bit later the likelihood-based method by which this was done. Equivalently, the pdf of the quasi-true dose $Y_{i}=f_{i}Q_{i}^{tr}/M_{i}^{mes}$ is given by:

$P[Y_{i}]=\sum_{k=1}^{K} \frac{p_{k}}{ \sigma_{k}\sqrt{2\pi}Y_{i}}\text{exp}\left[-\frac{[\ln(Y_{i})-\ln(\mu_{k})]^{2}}{2\sigma_{k}^{2}}\right]$ (S9)

Notice that:

$D_{i}^{mes}=f_{i}Q_{i}^{mes}/M_{i}^{mes}=(f_{i}Q_{i}^{tr}/M_{i}^{mes})V_{i}^{Q}=Y_{i}V_{i}^{Q}$ (S10)

Based on (S9) and (S10) the joint pdf of $(D_{i}^{mes},Y_{i})$ is given by:

$\begin{matrix} P[D_{i}^{mes},Y_{i}]=\frac{1}{\sigma_{Q,i} 2\pi D_{i}^{mes}Y_{i}}\text{exp}\left[-\frac{[\ln(D_{i}^{mes})-\ln(Y_{i})]^{2}}{2\sigma_{Q,i}^{2}}\right]\text{x} \\ \sum_{k=1}^{K} \frac{p_{k}}{ \sigma_{k}}\text{exp}\left[-\frac{[\ln(Y_{i})-\ln(\mu_{k})]^{2}}{2\sigma_{k}^{2}}\right] \end{matrix}$ (S11)

From (S10) this implies that the unconditional distribution of measured dose, $D_{i}^{mes}$, has pdf:

$P[D_{i}^{mes}]=\frac{1}{\sqrt{2\pi}D_{i}^{mes}}\sum_{k=1}^{K} \frac{p_{k}}{ \sqrt{\sigma_{Q,i}^{2}+\sigma_{k}^{2}}}\text{exp}\left[-\frac{[\ln(D_{i}^{mes})-\ln(\mu_{k})]^{2}}{2[\sigma_{Q,i}^{2}+\sigma_{k}^{2}]}\right]$ (S12)

i.e., a weighted sum of log-normal densities. From this expression the various unknown population parameters ($(\mu_{k},\sigma_{k})_{k=1}^{K}$) can be derived via maximum likelihood techniques, based on the distribution of observed dose in the sample and knowledge of the error distribution log standard deviation, $\sigma_{Q,i}$. By comparison, the second method assumes that the logarithm of $Q_{i}^{tr}$ has a distribution which is a sum of normal distributions, so that the pdf of $Q_{i}^{tr}$ is given by:

$P[Q_{i}^{tr}]=\sum_{k=1}^{K} \frac{p_{k}}{ \sigma_{k}\sqrt{2\pi}Q_{i}^{tr}}\text{exp}\left[-\frac{[\ln(Q_{i}^{tr})-\ln(\mu_{k})]^{2}}{2\sigma_{k}^{2}}\right]$ (SS9)

Based on (S3) and (SS9) the joint pdf of $(Q_{i}^{tr},Q_{i}^{mes})$ is given by:

$\begin{matrix} P[Q_{i}^{tr},Q_{i}^{mes}]=\frac{1}{\sigma_{Q,i} 2\pi Q_{i}^{mes}Q_{i}^{tr}}\text{exp}\left[-\frac{[\ln(Q_{i}^{mes})-\ln(Q_{i}^{tr})]^{2}}{2\sigma_{Q,i}^{2}}\right]\text{x} \\ \sum_{k=1}^{K} \frac{p_{k}}{ \sigma_{k}}\text{exp}\left[-\frac{[\ln(Q_{i}^{tr})-\ln(\mu_{k})]^{2}}{2\sigma_{k}^{2}}\right] \end{matrix}$ (SS11)

The second method (expression (SS11)) then implies that the unconditional distribution of measured activity has pdf:

$P[Q_{i}^{mes}]=\frac{1}{\sqrt{2\pi}Q_{i}^{mes}}\sum_{k=1}^{K} \frac{p_{k}}{ \sqrt{\sigma_{Q,i}^{2}+\sigma_{k}^{2}}}\text{exp}\left[-\frac{[\ln(Q_{i}^{mes})-\ln(\mu_{k})]^{2}}{2[\sigma_{Q,i}^{2}+\sigma_{k}^{2}]}\right]$ (SS12)

i.e., a weighted sum of log-normal densities. Formally, apart from a change of notation ($Q_{i}^{mes}$for $D_{i}^{mes}$, $Q_{i}^{tr}$for $Y_{i}$), these are the same equations as (S9), (S11) and (S12). As above, from (SS12) the various unknown population parameters ($(\mu_{k},\sigma_{k})_{k=1}^{K}$) can be derived via maximum likelihood techniques, based on the distribution of observed activity measurements in the sample. From (S11) and (S12) we derive the conditional distribution of $Y_{i}=f_{i}Q_{i}^{tr}/M_{i}^{mes}$ given $D_{i}^{mes}$, which has pdf:

$P[Y_{i}|D_{i}^{mes}]=\frac{\sum_{k=1}^{K} \left\{\begin{matrix} \frac{\sqrt{\sigma_{k}^{2}+\sigma_{Q,i}^{2}}}{ Y_{i}\sigma_{k}\sigma_{Q,i}\sqrt{2\pi}}\text{exp}\left[-\frac{\left[\begin{matrix} \ln(Y_{i})- \\ \frac{[\ln(\mu_{k})\sigma_{Q,i}^{2}+\ln(D_{i}^{mes})\sigma_{k}^{2}]}{[\sigma_{k}^{2}+\sigma_{Q,i}^{2}]} \end{matrix}\right]^{2}}{2\left(\sigma_{k}^{2}\sigma_{Q,i}^{2}/[\sigma_{k}^{2}+\sigma_{Q,i}^{2}]\right)}\right]\text{x} \\ \frac{p_{k}}{ D_{i}^{mes}\sqrt{\sigma_{k}^{2}+\sigma_{Q,i}^{2}}\sqrt{2\pi}}\text{exp}\left[-\frac{[\ln(D_{i}^{mes})-\ln(\mu_{k})]^{2}}{2[\sigma_{k}^{2}+\sigma_{Q,i}^{2}]}\right] \end{matrix}\right\}}{\sum_{k=1}^{K} \left\{\frac{p_{k}}{D_{i}^{mes}\sqrt{\sigma_{k}^{2}+\sigma_{Q,i}^{2}}\sqrt{2\pi}}\text{exp}\left[-\frac{[\ln(D_{i}^{mes})-\ln(\mu_{k})]^{2}}{2[\sigma_{k}^{2}+\sigma_{Q,i}^{2}]}\right]\right\}}$ (S13)

In particular:

$E[Y_{i}|D_{i}^{mes}]=\frac{\sum_{k=1}^{K} \left\{\begin{matrix} \exp\left[\frac{[\ln(\mu_{k})\sigma_{Q,i}^{2}+\ln(D_{i}^{mes})\sigma_{k}^{2}]+0.5\sigma_{k}^{2}\sigma_{Q,i}^{2}}{[\sigma_{k}^{2}+\sigma_{Q,i}^{2}]}\right]\text{x} \\ \frac{p_{k}}{ \sqrt{\sigma_{k}^{2}+\sigma_{Q,i}^{2}}}\text{exp}\left[-\frac{[\ln(D_{i}^{mes})-\ln(\mu_{k})]^{2}}{2[\sigma_{k}^{2}+\sigma_{Q,i}^{2}]}\right] \end{matrix}\right\}}{\sum_{k=1}^{K} \left\{\frac{p_{k}}{\sqrt{\sigma_{k}^{2}+\sigma_{Q,i}^{2}}}\text{exp}\left[-\frac{[\ln(D_{i}^{mes})-\ln(\mu_{k})]^{2}}{2[\sigma_{k}^{2}+\sigma_{Q,i}^{2}]}\right]\right\}}$ (S14)

By contrast, the second method ((SS9) and (SS11)) implies that the conditional distribution of the true activity given the measured has pdf:

$P[Q_{i}^{tr}|Q_{i}^{mes}]=\frac{\sum_{k=1}^{K} \left\{\begin{matrix} \frac{\sqrt{\sigma_{k}^{2}+\sigma_{Q,i}^{2}}}{ Q_{i}^{tr}\sigma_{k}\sigma_{Q,i}\sqrt{2\pi}}\text{exp}\left[-\frac{\left[\begin{matrix} \ln(Q_{i}^{tr})- \\ \frac{[\ln(\mu_{k})\sigma_{Q,i}^{2}+\ln(Q_{i}^{mes})\sigma_{k}^{2}]}{[\sigma_{k}^{2}+\sigma_{Q,i}^{2}]} \end{matrix}\right]^{2}}{2\left(\sigma_{k}^{2}\sigma_{Q,i}^{2}/[\sigma_{k}^{2}+\sigma_{Q,i}^{2}]\right)}\right]\text{x} \\ \frac{p_{k}}{ Q_{i}^{mes}\sqrt{\sigma_{k}^{2}+\sigma_{Q,i}^{2}}\sqrt{2\pi}}\text{exp}\left[-\frac{[\ln(Q_{i}^{mes})-\ln(\mu_{k})]^{2}}{2[\sigma_{k}^{2}+\sigma_{Q,i}^{2}]}\right] \end{matrix}\right\}}{\sum_{k=1}^{K} \left\{\frac{p_{k}}{ Q_{i}^{mes}\sqrt{\sigma_{k}^{2}+\sigma_{Q,i}^{2}}\sqrt{2\pi}}\text{exp}\left[-\frac{[\ln(Q_{i}^{mes})-\ln(\mu_{k})]^{2}}{2[\sigma_{k}^{2}+\sigma_{Q,i}^{2}]}\right]\right\}}$ (SS13)

It then follows that:

$E[Q_{i}^{tr}|Q_{i}^{mes}]=\frac{\sum_{k=1}^{K} \left\{\begin{matrix} \text{exp}\left[\frac{\ln(\mu_{k})\sigma_{Q,i}^{2}+\ln(Q_{i}^{mes})\sigma_{k}^{2}+0.5\sigma_{k}^{2}\sigma_{Q,i}^{2}}{[\sigma_{k}^{2}+\sigma_{Q,i}^{2}]}\right]\text{x} \\ \frac{p_{k}}{ \sqrt{\sigma_{k}^{2}+\sigma_{Q,i}^{2}}}\text{exp}\left[-\frac{[\ln(Q_{i}^{mes})-\ln(\mu_{k})]^{2}}{2[\sigma_{k}^{2}+\sigma_{Q,i}^{2}]}\right] \end{matrix}\right\}}{\sum_{k=1}^{K} \left\{\frac{p_{k}}{\sqrt{\sigma_{k}^{2}+\sigma_{Q,i}^{2}}}\text{exp}\left[-\frac{[\ln(Q_{i}^{mes})-\ln(\mu_{k})]^{2}}{2[\sigma_{k}^{2}+\sigma_{Q,i}^{2}]}\right]\right\}}$ (SS14)

Again, formally, apart from a change of notation ($Q_{i}^{mes}$for $D_{i}^{mes}$, $Q_{i}^{tr}$for $Y_{i}$), these are the same equations as (S13) and (S14). Notice that:

$D_{i}^{tr}=f_{i}Q_{i}^{tr}/M_{i}^{tr}=(f_{i}Q_{i}^{tr}/M_{i}^{mes})/V_{i}^{M}=Y_{i}/V_{i}^{M}$ (S15)

By assumption, $Y_{i},V_{i}^{M}$ are independent, so that by (S6):

$$\begin{matrix} E[D_{i}^{tr}|D_{i}^{mes}]=E[Y_{i}/V_{i}^{M}|D_{i}^{mes}]=E[Y_{i}|D_{i}^{mes}]E[1/V_{i}^{M}] \\ =\exp\left[\frac{\sigma_{M,i}^{2}}{2}\right]\frac{\sum_{k=1}^{K} \left\{\begin{matrix} \exp\left[\frac{\ln(\mu_{k})\sigma_{Q,i}^{2}+\ln(D_{i}^{mes})\sigma_{k}^{2}+0.5\sigma_{k}^{2}\sigma_{Q,i}^{2}}{[\sigma_{k}^{2}+\sigma_{Q,i}^{2}]}\right]\text{x} \\ \frac{p_{k}}{ D_{i}^{mes}\sqrt{\sigma_{k}^{2}+\sigma_{Q,i}^{2}}\sqrt{2\pi}}\text{exp}\left[-\frac{[\ln(D_{i}^{mes})-\ln(\mu_{k})]^{2}}{2[\sigma_{k}^{2}+\sigma_{Q,i}^{2}]}\right] \end{matrix}\right\}}{\sum_{k=1}^{K} \left\{\frac{p_{k}}{D_{i}^{mes}\sqrt{\sigma_{k}^{2}+\sigma_{Q,i}^{2}}\sqrt{2\pi}}\text{exp}\left[-\frac{[\ln(D_{i}^{mes})-\ln(\mu_{k})]^{2}}{2[\sigma_{k}^{2}+\sigma_{Q,i}^{2}]}\right]\right\}} \end{matrix}$$

$=\exp\left[\frac{\sigma_{M,i}^{2}}{2}\right]\frac{\sum_{k=1}^{K} \left\{\begin{matrix} \exp\left[\frac{\ln(\mu_{k})\sigma_{Q,i}^{2}+\ln(D_{i}^{mes})\sigma_{k}^{2}+0.5\sigma_{k}^{2}\sigma_{Q,i}^{2}}{[\sigma_{k}^{2}+\sigma_{Q,i}^{2}]}\right]\text{x} \\ \frac{p_{k}}{ \sqrt{\sigma_{k}^{2}+\sigma_{Q,i}^{2}}}\text{exp}\left[-\frac{[\ln(D_{i}^{mes})-\ln(\mu_{k})]^{2}}{2[\sigma_{k}^{2}+\sigma_{Q,i}^{2}]}\right] \end{matrix}\right\}}{\sum_{k=1}^{K} \left\{\frac{p_{k}}{\sqrt{\sigma_{k}^{2}+\sigma_{Q,i}^{2}}}\text{exp}\left[-\frac{[\ln(D_{i}^{mes})-\ln(\mu_{k})]^{2}}{2[\sigma_{k}^{2}+\sigma_{Q,i}^{2}]}\right]\right\}}$ (S16)

By contrast, the second method estimates:

$\begin{matrix} E[D_{i}^{tr}|Q_{i}^{mes},M_{i}^{mes}]=E[f_{i}Q_{i}^{tr}/M_{i}^{tr}|Q_{i}^{mes},M_{i}^{mes}] \\ =f_{i}E[Q_{i}^{tr}|Q_{i}^{mes}]E[1/M_{i}^{tr}|M_{i}^{mes}] \end{matrix}$ (SS15)

(making use of the independence of $(Q_{i}^{tr},V_{i}^{Q},M_{i}^{mes},V_{i}^{M})$ in the second equality) and (S6), (SS14) and (SS15) therefore imply that the conditional expectation of the true dose given all the measured quantities ($Q_{i}^{mes},M_{i}^{mes}$) is:

$E[D_{i}^{tr}|Q_{i}^{mes},M_{i}^{mes}]=f_{i}\frac{\exp\left[\frac{\sigma_{M,i}^{2}}{2}\right]}{M_{i}^{mes}}\frac{\sum_{k=1}^{K} \left\{\begin{matrix} \text{exp}\left[\frac{\ln(\mu_{k})\sigma_{Q,i}^{2}+\ln(Q_{i}^{mes})\sigma_{k}^{2}+0.5\sigma_{k}^{2}\sigma_{Q,i}^{2}}{[\sigma_{k}^{2}+\sigma_{Q,i}^{2}]}\right]\text{x} \\ \frac{p_{k}}{ \sqrt{\sigma_{k}^{2}+\sigma_{Q,i}^{2}}}\text{exp}\left[-\frac{[\ln(Q_{i}^{mes})-\ln(\mu_{k})]^{2}}{2[\sigma_{k}^{2}+\sigma_{Q,i}^{2}]}\right] \end{matrix}\right\}}{\sum_{k=1}^{K} \left\{\frac{p_{k}}{\sqrt{\sigma_{k}^{2}+\sigma_{Q,i}^{2}}}\text{exp}\left[-\frac{[\ln(Q_{i}^{mes})-\ln(\mu_{k})]^{2}}{2[\sigma_{k}^{2}+\sigma_{Q,i}^{2}]}\right]\right\}}$ (SS16)

**Note:** Kukush *et al.* [13] state that “the sample correlation between $\log(Q_{i}^{tr})$ and $\log(M_{i}^{mes})$ is equal to 0.26.” In that paper for the underlying radio-epidemiological study, the sample correlation between $\ln(Q_{i}^{mes})$ and $\log\left( Q_{i}^{\mathrm{mes}} \right)and log(M_{i}^{\mathrm{mes}})$ was reported, because in simulations made in Kukush *et al.* [13], the actual values of $Q_{i}^{mes}$ were taken as $Q_{i}^{tr}$ and classical multiplicative error was imposed on those values. In our data, we found no evidence for correlation between $Q_{i}^{mes}$ and $M_{i}^{mes}$: we estimated the Pearson correlation coefficient between them to be about -0.05, implying that they are largely uncorrelated. As such, there is no evidence to invalidate use of the second regression calibration model, although the first model, which does not make such strong assumptions must be regarded as *a priori* more plausible.

**Fitting of thyroid cancer risk model to data**

An excess odds ratio (EOR) model was employed, in which the probability of thyroid cancer for an individual with age $a$ at screening, age $e$ at exposure, with true thyroid dose $D_{i}^{tr}=f_{i}Q_{i}^{tr}/M_{i}^{tr}$ of gender $s$ is given by:

$\begin{matrix} P(a,e,s,D|\alpha,\beta,\gamma,\kappa,\tau,\psi) \\ =\frac{\exp\left[\beta_{0}+\beta_{s}1_{s=male}+\sum_{k=1}^{N} \beta_{k}1_{a_{k-1}\leq a<a_{k}}\right]\left[1+\alpha D\exp\left(\gamma D+\kappa[e-8]+\tau[a-22]+\psi[a-e-14]+\eta1_{s=male}\right)\right]}{1+\exp\left[\beta_{0}+\beta_{s}1_{s=male}+\sum_{k=1}^{N} \beta_{k}1_{a_{k-1}\leq a<a_{k}}\right]\left[1+\alpha D\exp\left(\gamma D+\kappa[e-8]+\tau[a-22]+\psi[a-e-14]+\eta1_{s=male}\right)\right]} \end{matrix}$ (S17)

Therefore the likelihood is given by:

$\begin{matrix} L(\alpha,\beta,\gamma,\kappa,\tau,\psi|a_{i},e_{i},s_{i},D_{i}^{tr},\delta_{i})=L(\alpha,\beta,\gamma,\kappa,\tau,\psi|a_{i},e_{i},s_{i},Q_{i}^{tr},M_{i}^{tr},\delta_{i}) \\ =\prod_{i=1}^{N} P(a_{i},e_{i},s_{i},D_{i}^{tr}|\alpha,\beta,\gamma,\kappa,\tau,\psi)^{\delta_{i}}\left[1-P(a_{i},e_{i},s_{i},D_{i}^{tr}|\alpha,\beta,\gamma,\kappa,\tau,\psi)\right]^{1-\delta_{i}} \end{matrix}$ (S18)

# where $\delta_{i}$ is the indicator of whether person $i$ was a case (=1 if so, =0 otherwise). This of course is a function of unknown variables $f_{i},M_{i}^{tr},Q_{i}^{tr}$. For the reasons given by Fearn *et al.* [14] and Stram and Kopecky [12], it is sensible to consider instead the integrated likelihood, given by:

$\begin{matrix} E[L|M_{i}^{mes},Q_{i}^{mes},i=1,...,N] \\ =E\left[\prod_{i=1}^{N} P(a_{i},e_{i},s_{i},D_{i}^{tr}|\alpha,\beta,\gamma,\kappa,\tau,\psi)^{\delta_{i}}\left[1-P(a_{i},e_{i},s_{i},D_{i}^{tr}|\alpha,\beta,\gamma,\kappa,\tau,\psi)\right]^{1-\delta_{i}}|M_{i}^{mes},Q_{i}^{mes},i=1,...,N\right] \\ =\prod_{i=1}^{N} E\left[\prod_{i=1}^{N} P(a_{i},e_{i},s_{i},D_{i}^{tr}|\alpha,\beta,\gamma,\kappa,\tau,\psi)^{\delta_{i}}\left[1-P(a_{i},e_{i},s_{i},D_{i}^{tr}|\alpha,\beta,\gamma,\kappa,\tau,\psi)\right]^{1-\delta_{i}}|M_{i}^{mes},Q_{i}^{mes}\right] \end{matrix}$ (S19)

which can be integrated via Monte Carlo sampling from the distributions (S5) and (S13) (or (SS13)) (and noting (S15)). We use Monte Carlo integration in addition to the two regression calibration methods. For the purposes of this paper, we substitute $D_{i}^{tr}$ by $E[D_{i}^{tr}|D_{i}^{mes}]$ for the first regression calibration method (adapted from Kukush *et al.* [13]) as outlined above, or $D_{i}^{tr}$ by $E[D_{i}^{tr}|Q_{i}^{mes},M_{i}^{mes}]$ as in the second method, in (S18). Whether using these two regression calibration approaches or Monte Carlo integration, the model parameters $\alpha,\beta,\gamma,\kappa,\tau$ are estimated via maximizing the logistic likelihood [21]. In general, because of collinearity in the age and temporal variables (age $a$ at screening, age $e$ at exposure, attained age $a+e$), only one of the age or temporal adjustment parameters, $\kappa$, $\tau$ or $\psi$ was free to vary. The Monte Carlo sample of doses was generated by various of the study team (VMS, IAL).

**Preliminary model fits to dose model**

As can be seen from Supporting Information Table S1, there is evidence that a combination of three log-normal models are required to fit the measured dose data, using expression (S12). With the addition of any model up to the third there is a significant improvement in fit (*p*<0.03), but larger numbers of log-normal distributions yield no significant improvement in fit (*p*>0.5). The log-likelihood is virtually identical for models with three to seven log-normal distributions. Supporting Information Figure S3 demonstrates that the dose is distributed very-nearly log-normally. Therefore for all analyses using the first method, a combination of three log-normal distributions were assumed. Likewise, and as can be seen from Supporting Information Table S2, the second method modeled the true activity distribution using a scaled sum of three log-normal distributions. With the addition of any model up to the third there is a significant improvement in fit (*p*<0.001), but larger numbers of log-normal distributions yield no significant improvement in fit (*p*>0.1). [It should be noted that this is therefore slightly different from the approach adopted by Kukush *et al.* [13], who only used a single normal distribution. However, the theoretical simulations of Kukush *et al.* [13] were in effect a proof of principle, not requiring modeling of real data.]
