# Supplementary material for: Impact of Uncertainties in Exposure Assessment on Estimates of Thyroid Cancer Risk among Ukrainian Children and Adolescents Exposed from the Chernobyl Accident
Source: PLoS One. 2014 Jan 29;9(1):e85723. doi: 10.1371/journal.pone.0085723 (PMC3906013; doi:10.1371/journal.pone.0085723)
Supplement: Table S1 — Fits of multiple log-normal models as given by expression (S12) to measured dose data (1st regression calibration model). (DOCX) [file pone.0085723.s005.docx]

**Supporting Information Table S1. Fits of multiple log-normal models as given by expression (S12) to measured dose data (1^st^ regression calibration model).**

|  | 1 Normal model | 2 Normal models | 3 Normal models | 4 Normal models | 5 Normal models | 6 Normal models | 7 Normal models |
| --- | --- | --- | --- | --- | --- | --- | --- |
| Log likelihood | -11305.07 | -11240.64 | -11235.93 | -11235.61 | -11233.70 | -11233.70 | -11233.70 |
|  | *p*-value for improvement in fit | | | | | | |
|  | - | <0.001 | 0.024 | 0.888 | 0.280 | 1.000 | 1.000 |
| Parameter | Coefficients | | | |  |  |  |
| *σ*_1_ | 1.448 | 1.021 | <0.001 | <0.001 | <0.001 | <0.001 | <0.001 |
| *σ*_2_ | - | 1.294 | 0.931 | 1.059 | 1.097 | 1.097 | 1.097 |
| *σ*_3_ | - | - | 1.313 | 1.084 | 0.904 | 0.903 | 0.902 |
| *σ*_4_ | - | - | - | 0.350 | 0.424 | 0.424 | 0.424 |
| *σ*_5_ | - | - | - | - | 0.173 | 0.172 | 0.172 |
| *σ*_6_ | - | - | - | - | - | 0.922 | 0.927 |
| *σ*_7_ | - | - | - | - | - | - | 0.905 |
| *μ*_1_ | 0.186 | 0.074 | 0.007 | 0.261 | 0.263 | 0.263 | 0.263 |
| *μ*_2_ | - | 0.384 | 0.068 | 0.088 | 0.100 | 0.100 | 0.100 |
| *μ*_3_ | - | - | 0.345 | 0.635 | 0.808 | 0.805 | 0.800 |
| *μ*_4_ | - | - | - | 14.146 | 11.776 | 11.779 | 11.778 |
| *μ*_5_ | - | - | - | - | 4.778 | 4.779 | 4.780 |
| *μ*_6_ | - | - | - | - | - | 0.993 | 1.039 |
| *μ*_7_ | - | - | - | - | - | - | 1.026 |
| *p*_1_ | 1.000 | 0.435 | 0.006 | 0.012 | 0.015 | 0.015 | 0.015 |
| *p*_2_ | - | 0.565 | 0.364 | 0.617 | 0.704 | 0.703 | 0.704 |
| *p*_3_ | - | - | 0.630 | 0.368 | 0.271 | 0.265 | 0.259 |
| *p*_4_ | - | - | - | 0.002 | 0.005 | 0.005 | 0.005 |
| *p*_5_ | - | - | - | - | 0.005 | 0.005 | 0.005 |
| *P*_6_ | - | - | - | - | - | 0.006 | 0.006 |
| *P*_7_ | - | - | - | - | - | - | 0.006 |
