# Supplementary material for: Impact of Uncertainties in Exposure Assessment on Estimates of Thyroid Cancer Risk among Ukrainian Children and Adolescents Exposed from the Chernobyl Accident
Source: PLoS One. 2014 Jan 29;9(1):e85723. doi: 10.1371/journal.pone.0085723 (PMC3906013; doi:10.1371/journal.pone.0085723)
Supplement: Table S2 — Fits of multiple log-normal models to measured activity data (2nd regression calibration model). (DOCX) [file pone.0085723.s006.docx]

**Supporting Information Table S2. Fits of multiple log-normal models to measured activity data (2^nd^ regression calibration model).**

|  | 1 Normal model | 2 Normal models | 3 Normal models | 4 Normal models | 5 Normal models | 6 Normal models | 7 Normal models |
| --- | --- | --- | --- | --- | --- | --- | --- |
| Log likelihood | -11706.80 | -11552.25 | -11538.99 | -11538.99 | -11536.10 | -11536.04 | -11536.04 |
|  | *p*-value for improvement in fit | | | | | | |
|  | - | <0.001 | <0.001 | 1.000 | 0.123 | 0.990 | 1.000 |
| Parameter | Coefficients | | | |  |  |  |
| *σ*_1_ | 1.479 | 1.399 | 0.903 | 0.902 | 0.877 | 0.894 | 0.894 |
| *σ*_2_ | - | 0.979 | 0.948 | 0.949 | 1.170 | 1.167 | 1.167 |
| *σ*_3_ | - | - | 0.873 | 0.879 | 0.894 | 0.907 | 0.902 |
| *σ*_4_ | - | - | - | 0.873 | 0.132 | 0.000 | 0.000 |
| *σ*_5_ | - | - | - | - | 0.828 | 0.362 | 0.361 |
| *σ*_6_ | - | - | - | - | - | 0.806 | 0.806 |
| *σ*_7_ | - | - | - | - | - | - | 0.806 |
| *μ*_1_ | 6.450 | 14.060 | 84.600 | 84.779 | 97.520 | 92.041 | 92.091 |
| *μ*_2_ | - | 2.825 | 11.618 | 11.609 | 8.154 | 7.727 | 7.729 |
| *μ*_3_ | - | - | 2.251 | 2.255 | 2.273 | 2.319 | 2.320 |
| *μ*_4_ | - | - | - | 2.250 | 2.005 | 1.985 | 1.985 |
| *μ*_5_ | - | - | - | - | 1.774 | 2.152 | 2.151 |
| *μ*_6_ | - | - | - | - | - | 1.635 | 1.635 |
| *μ*_7_ | - | - | - | - | - | - | 1.636 |
| *p*_1_ | 1.000 | 0.521 | 0.093 | 0.093 | 0.069 | 0.076 | 0.076 |
| *p*_2_ | - | 0.479 | 0.444 | 0.444 | 0.670 | 0.691 | 0.691 |
| *p*_3_ | - | - | 0.463 | 0.000 | 0.000 | 0.000 | 0.000 |
| *p*_4_ | - | - | - | 0.463 | 0.020 | 0.011 | 0.011 |
| *p*_5_ | - | - | - | - | 0.241 | 0.019 | 0.019 |
| *P*_6_ | - | - | - | - | - | 0.204 | 0.086 |
| *P*_7_ | - | - | - | - | - | - | 0.118 |
