# Supplementary material for: Impact of Uncertainties in Exposure Assessment on Estimates of Thyroid Cancer Risk among Ukrainian Children and Adolescents Exposed from the Chernobyl Accident
Source: PLoS One. 2014 Jan 29;9(1):e85723. doi: 10.1371/journal.pone.0085723 (PMC3906013; doi:10.1371/journal.pone.0085723)
Supplement: Table S3 — Effect of additional background variables on thyroid cancer prevalence risk (EOR/Gy). All use a linear EOR model, with regression calibration dose adjustments adapted from Kukush et al [13]. All CI are profile-likelihood based. (DOCX) [file pone.0085723.s007.docx]

**Supporting Information Table S3. Effect of additional background variables on thyroid cancer prevalence risk (EOR/Gy).** All use a linear EOR model, with regression calibration dose adjustments adapted from Kukush *et al* [13]. All CI are profile-likelihood based.

| Background model | *p*-value for improvement in fit over baseline model (with age at screening and sex in background) | EOR / Gy (+95% CI) |
| --- | --- | --- |
| Age + sex (baseline) | - | 5.78 (1.92, 27.04) |
| Age + sex + marital status | 0.100 | 5.52 (1.84, 25.12) |
| Age + sex + urban status | 0.727 | 5.46 (1.81, 24.97) |
| Age + sex + oblast | 0.715 | 8.77 (2.45, 72.71) |
| Age + sex + goiter | 0.203 | 5.57 (1.85, 25.51) |
| Age + sex + other thyroid pathology | 0.713 | 5.72 (1.90, 26.63) |
| Age + sex + thyroid cancer in relatives | 0.671 | 5.84 (1.94, 27.49) |
| Age + sex + goitre in relatives | 0.729 | 5.82 (1.93, 27.33) |
| Age + sex + other thyroid pathology in relatives | 0.520 | 5.86 (1.95, 27.62) |
